# Dual QM and MM Approach for Computing Equilibrium Isotope Fractionation Factor of Organic Species in Solution

**DOI:** 10.3390/molecules23102644

**Published:** 2018-10-15

**Authors:** Meiyi Liu, Katelyn N. Youmans, Jiali Gao

**Affiliations:** 1 Laboratory of Theoretical and Computational Chemistry, Theoretical Chemistry Institute, Jilin University, Changchun 130023, China; liumy13@mails.jlu.edu.cn; 2 Department of Chemistry and Supercomputing Institute, University of Minnesota, Minneapolis, MN 55455, USA; youm0013@umn.edu

**Keywords:** combined QM/MM, path integral simulation, PI-FEP, equilibrium isotope effects, nuclear quantum effects

## Abstract

A dual QM and MM approach for computing equilibrium isotope effects has been described. In the first partition, the potential energy surface is represented by a combined quantum mechanical and molecular mechanical (QM/MM) method, in which a solute molecule is treated quantum mechanically, and the remaining solvent molecules are approximated classically by molecular mechanics. In the second QM/MM partition, differential nuclear quantum effects responsible for the isotope effect are determined by a statistical mechanical double-averaging formalism, in which the nuclear centroid distribution is sampled classically by Newtonian molecular dynamics and the quantum mechanical spread of quantized particles about the centroid positions is treated using the path integral (PI) method. These partitions allow the potential energy surface to be properly represented such that the solute part is free of nuclear quantum effects for nuclear quantum mechanical simulations, and the double-averaging approach has the advantage of sampling efficiency for solvent configuration and for path integral convergence. Importantly, computational precision is achieved through free energy perturbation (FEP) theory to alchemically mutate one isotope into another. The PI-FEP approach is applied to model systems for the ^18^O enrichment found in cellulose of trees to determine the isotope enrichment factor of carbonyl compounds in water. The present method may be useful as a general tool for studying isotope fractionation in biological and geochemical systems.

## 1. Introduction

Stable isotope compositions in plants and organisms are sensitive to the environment in which biosynthesis takes place. Analyses of the ^18^O enrichment of cellulose in tree rings could reveal historical records of local climate and ecohydrological changes such as temperature, humidity, and precipitation [1,2,3,4]. Isotope abundances of light elements are reported as the relative ratio of the heavy to light isotope of the sample with respect to a standard of known composition [5]. For example, the ^18^O isotope enrichment of a biomass is given by

(1) $$\delta^{18}O=1000\times (\frac{R_{sample}}{R_{\mathrm{VSMOW}}}-1)$$

where $R={[}^{18}O]/{[}^{16}O]$ is an isotope ratio, and VSMOW (Vienna standard mean ocean water) is the international standard for oxygen and hydrogen. Another commonly used term is the equilibrium isotope fractionation factor of different compounds *A* and *B*, or the same compounds in two different media (also indicated by *A* and *B*), denoted by $\alpha_{A/B}=R_{A}/R_{B}$. Isotope fractionation factor is directly related to the *δ* values in Equation (1) by $\alpha_{A/B}=(\delta A+1000)/(\delta B+1000)$). Thus, for example, the enrichment of ^18^O of acetone in water can be described by the isotope fractionation factor between the carbonyl compound and water in aqueous solution [6]. Remarkably, there is a global average $\delta^{18}O$ of 27‰ enrichment in cellulose of trees relative to the source water during its biosynthesis [7]. However, correlation between isotope signature and climate parameters could be challenging since many dynamic, thermodynamic, and kinetic factors can influence the final composition [4], including the isotope signal of stem water reflecting precipitation, the ^18^O enrichment of leaf water due to evaporation that is sensitive to environmental temperature and humidity [8], and biochemical fractionation during carbohydrate and cellulose biosynthesis [9]. A primary biochemical origin responsible for the ^18^O fractionation of cellulose is the equilibrium isotope effects between carbonyl groups and water during cellulose biosynthesis [6,10]. Thus, it is of interest to develop and test a computational method for estimating isotope fractionation under different environmental conditions in solution and in biological systems.

Isotope fractionation reports nuclear quantum effects (NQE) since the potential energy surface determined by the electronic structure is independent of atomic mass [11]. On the other hand, the chemical equilibria of the different isotopologues are critically dependent on interactions with solvent molecules and the environment in which the isotope exchange occurs. Thus, it is of interest to understand the interplay of NQE and intermolecular interactions on the observed isotope effects in a given environment. The purpose of this study is to describe a molecular dynamics simulation approach that integrates Feynman path integral with free energy perturbation (PI-FEP) calculations to determine equilibrium isotope fractionation factor in condensed phases. The method has been developed previously for studying kinetic isotope effects of enzyme-catalyzed reactions. Here, we make use of acetaldehyde and acetone in water as model compounds for the carbonyl groups of carbohydrates to illustrate the PI-FEP method for equilibrium isotope fractionation.

Three important factors—potential, precision, and efficiency—need to be considered in calculation of the small free energy difference due to NQE resulting from isotope substitutions. First, the potential energy surface governing nuclear motions of the solute molecule, i.e., the species in consideration for isotope fractionation, must be “free of NQE”. Standard effective potentials developed for liquids and biomolecular simulations are not appropriate since these empirical potential energy functions incorporate implicitly NQE (and polarization for pairwise potentials) in the parameterization process by reproducing experimental data (e.g., heat of vaporization and density) [12]. Yet, accurate gas-phase potentials do not account for solute–solvent interactions [13]. On the other hand, empirical effective potentials can be used (also desired to include solvent NQE implicitly) to describe intermolecular interactions of the solvent for computational efficiency in dynamics simulations. Of course, one could treat nuclear quantum effects both for the solute and solvent on an equal footing as in ab initio molecular dynamics, but this limits potential applications to small systems. A combined quantum mechanical and molecular mechanical (QM/MM) potential [14,15] fits this demand nicely since the electronic structure of the solute molecule provides a true Born–Oppenheimer potential energy surface. At the same time, it incorporates the effective “external” potential of the environment through QM/MM interactions. Another important consideration is to properly treat the change of the potential energy surface for the solute molecule due to instantaneous fluctuations of the solvent molecules [16]. This is naturally included in a QM/MM potential, whereas it would be difficult, if not impossible, to parameterize empirical functions that change form (and parameters) in response to solvent fluctuations in general. The use of a combined QM/MM method to model the potential energy surface is the first partition of a system into QM and MM regions.

Secondly, it is essential to develop a procedure to provide the necessary precision to determine the exceedingly small (with respect to fluctuations of kinetic energy) free energy difference due to NQE responsible for the observed isotope fractionation of organic compounds in solution or in biosynthesis [17]. The change in zero-point energy due to molecular vibrations of isotope exchange undoubtedly makes a dominant contribution to $\alpha_{A/B}$, though other nuclear quantum effects also contribute. In this context, the use of harmonic vibrational frequencies of isolated molecules in vacuum based on optimized geometries may not be sufficient for estimating isotope effects. Here, it is important to include anharmonic contributions. Further, isotope fractionation is concerned with the effects of environment on chemical equilibria. Consequently, it is desirable to employ statistical mechanical Monte Carlo and molecular dynamics simulations to determine the relative free energies and equilibrium constants. The theoretical framework in our approach is discrete path integral simulation, and, importantly, we achieve the required precision through free energy perturbation [18] of atomic masses in a single simulation [17]. Consequently, we obtain directly the ratio of the mass-dependent partition functions, i.e., the equilibrium constant, between the heavy (*H*) and light (*L*) isotopologues:

(2) $$R=\frac{\left[H\right]}{\left[L\right]}=\frac{Q_{H}^{qm}}{Q_{L}^{qm}}=\;<e^{-\beta \Delta V_{eff}^{L\to H}}{>}_{L}$$

where $\Delta V_{eff}^{L\to H}$ is the difference in the quantum-mechanical (*qm*) effective potential [19] between the light and heavy isotopes, $<\cdots {>}_{L}$ indicates an ensemble average over the potential for species *L*, and $\beta =1/k_{B}T$ with $k_{B}$ being Boltzmann’s constant and T the absolute temperature. We emphasize that the present path integral simulations are carried out for one isotopologue [17,20,21]—typically the most abundant one—which is the light particle of oxygen (^16^O) in the present study (of course, there is no difference if one chooses to carry out the simulation using the heavy particle). Note also that “*L*” and “*H*” in Equation (2) are not limited to a single isotope exchange, but multiple substitutions of different atoms can be simultaneously treated. 

The third consideration is concerned with efficiency of configuration sampling, to obtain converged results not only in path integral simulation, but also in solvent effects on NQE. This is particularly important when highly heterogeneous environments are encountered, such as the active site of a protein [17,22]. Both criteria are met in our algorithm, in which the quantum mechanical average $<\cdots {>}_{L}$ in Equation (2) is exactly separated into two simulation steps: one for configurational sampling of the entire system and one for the NQE of quantized nuclei. First, we carry out a classical mechanical, i.e., Newtonian molecular dynamics, simulation to generate an ensemble of nuclear coordinates. For the present solution phase system consisting of about 5000 particles, one can easily generate a 100-ns trajectory per day. Thus, convergence on solvent distribution can be adequately obtained. In the second computational step, convergence in NQE is achieved because we determine the relative NQE of the real system with respect to a known, free-particle reference state through free-particle path-integral (FPPI) sampling. In FPPI simulation, the probability density of a free particle distribution is known exactly [17,22,23]. Thus, different discretized paths are generated in a way completely independent of each other, avoiding to be trapped in local minima that can cause abrupt changes in PI average [24,25]. Therefore, the quantum mechanical average of a property, *O*, including the ratio of partition functions in Equation (2), can be rigorously determined as follows [19]

(3) $$<O{>}_{qm}\;=\;<<O{>}_{\mathrm{FP}}{>}_{\mathrm{CM}}\;$$

where the inner average $<\cdots {>}_{{}_{\mathrm{FP}}}$ indicates that the discrete path integral simulation is carried out using a free particle reference state, and the outer average $<\cdots {>}_{\mathrm{CM}}$ represents a classical mechanical ensemble average from molecular dynamics or statistical Monte Carlo simulations [17,26,27]. 

The separation of the quantum mechanical path integral simulation (Equation (3)) into a classical mechanical step and an FPPI sampling to account for quantum mechanical effects is the second partition of a condensed phase system into QM and MM regions. Thus, the present approach may be considered as a dual QM and MM technique for (1) the QM/MM representation of the potential energy surface and for (2) the QM-MM sampling of the nuclear dynamics motions. 

The remainder of the article will first address the three considerations in more detail in Section 2. Then, computational details are provided for determining isotope fractionation factors of acetone and acetaldehyde in water in Section 3. Results and discussion are presented in Section 4. The paper will conclude with a summary of the main findings from this study.

## 2. Methods

Almost all enzyme reactions and solutions can be well described by the Born–Oppenheimer approximation (except, for example, photochemical processes), in which the sum of the electronic energy and the nuclear repulsion provides a potential energy function, or potential energy surface (PES), governing the interatomic motions [28]. Therefore the molecular modeling problem breaks into two parts: the PES and the dynamics simulations. 

### 2.1. Potential Energy Surface

The potential energy function used in the present study follows a combined quantum mechanical and molecular mechanical (QM/MM) approach, in which the electronic structure of the solute molecule is described by a quantum chemical model and the rest of the solvent molecules are approximated by molecular mechanical force fields [14,29,30,31,32]. Standard procedures are used in this study, and a summary of modern QM/MM approaches to enable such a hybrid representation of the system to produce an accurate description of intermolecular interactions can be found in an early summary [14] and for a review of progresses in a previous work [15]. Although it is possible to treat an entire solute–solvent-enzyme system fully by a fragment-based quantum chemical method such as Hartree–Fock theory or Kohn–Sham density functional theory [33,34,35,36,37], the computational costs are still too expensive for routine free energy simulations that require extensive sampling. Thus, arguably, the most effective approach to model solvation and chemical reactions in enzymes remains a combined QM/MM model [15], which offers both computational efficiency and accuracy [14]. 

Although not used directly in the present study, we note that a particularly important approach to increasing computational accuracy is to develop dual-level QM/MM methods in which configurational sampling is performed with a low-level (*LL*), presumably computationally efficient, QM model for solute–solvent (*XS*) interactions ($\Delta E_{qm/mm}^{LL}(XS)$), followed by a high-level (HL) QM treatment of the solute, *X*, $E_{qm}^{HL}(X)$ to better represent the “QM” subsystem [38].

(4) $$U_{}^{DL}=E_{qm}^{HL}(X)+\Delta E_{qm/mm}^{LL}(XS)+U_{mm}(S)\;$$

where *X* and *S* denote solute and solvent coordinates, the subscripts specify the representation of the system in parentheses, and the superscripts indicate the level of theory used in energy calculation. Note that the acronyms, *LL* and *HL*, for the representation of the potential energy surfaces in this section should not be confused with heavy and light isotopes in the rest of the paper. The key concept in Equation (4) is to limit the time-demanding *HL* computation to the smaller part of the system, the solute, in the gas phase, while the computationally efficient *LL* model is applied to solute–solvent interactions for dynamics simulation. The latter must be repeated hundreds of million times (~100 ns time scale) for configurational sampling, essential for understanding solvent effects and the contributions of protein dynamics to catalysis. This is only possible using a relatively lower-level (*LL*) method for QM/MM interactions (but parameterized for an accurate description of solute–solvent interactions). Interested readers are directed to some recent studies in the literature [39,40,41,42,43,44]. 

One should keep in mind that any combined QM/MM potential is an empirical model that contains unavoidably empirical parameters [32,45]. Thus, it is useful to take advantage of the presence of these well-defined parameters to optimize them [32,46,47] to achieve the desired accuracy for describing solute–solvent interactions [32,45,48,49,50,51,52,53]. Note also that a molecular orbital method with electron correlation or a DFT QM/MM potential does not guarantee higher accuracy than Hartree–Fock theory or a semi-empirical method such as the Austin model 1 (AM1) or tight-binding density functional theory (DFTB) [54,55,56,57], if the empirical QM/MM parameters have not been optimized for the higher level of QM theory in use.

### 2.2. Path Integral-Free Energy Perturbation

The equilibrium constant, $R_{A}$, between different isotopologues of substance *A* (Equation (1)) is related to the free energy difference ($\Delta G_{A}^{L\to H}$) resulting from an isotope exchange, and it is directly computed by evaluating the ratio of their partition functions.

(5) $$R_{A}=e^{-\beta \Delta G_{A}^{L\to H}}=\frac{Q_{A}^{H}}{Q_{A}^{L}}$$

The QM partition function of the hybrid (quantized and classical nuclei) system $Q_{A}^{Y}$ (Y=H,L) by Feynman path integral [19,58,59] may be expressed as follows

(6) $$Q_{A}^{Y}=N^{Y}\int dS\int d{\bar{r}}_{A}^{Y}\oint D[r_{A}^{Y}]e^{-\beta V_{eff}(r_{A}^{Y},S)}\;$$

where $N^{Y}$ is a normalization factor, ${\bar{r}}_{A}^{Y}$ is the reference coordinates of all quantized particles, S denotes coordinates for the rest of the system, and $V_{eff}(r_{A}^{Y},S)$ is an effective quantum mechanical potential, and $\oint D[r_{A}^{Y}]\cdots$ denotes a path integral over all closed paths that begin and end at ${\bar{r}}_{A}^{Y}$ over the imaginary time that the path is traversed. 

If the path integration is discretized and represented by a ring of *P* quasi-particles called beads, the effective potential may be expressed as

(7) $$V_{eff}^{}(r_{A}^{Y},S)=\sum_{q=1}^{M}\frac{P}{2\beta \lambda_{q}^{2}}\sum_{i}^{P}{\left(r_{i}^{q}-r_{i+1}^{q}\right)}^{2}+\frac{1}{P}\sum_{i}^{P}U(r_{i}^{\left\{M\right\}},S)\;$$

where the path $r_{A}^{Y}$ for the *M*-quantized particles is collectively denoted by $r_{A}^{Y}=\{r_{i}^{q};i=1,\cdots ,P;$q=1,⋯,M} with the convention of $r_{P+1}=r_{1}$, $U(r_{i}^{\left\{M\right\}},S)$ is the QM/MM potential energy of the *i*th slice of the path, and $\lambda_{q}$ is the de Broglie thermal wavelength of a particle of mass $m_{q}$($\lambda_{q}^{2}=\beta \hbar^{2}/m_{q}$) with ℏ being Planck’s constant divided by 2π. In this work, the path integral over quantized nuclei in Equation (6) may be denoted by $\int d{\bar{r}}_{A}^{Y}\oint D[r_{A}^{Y}]=\int dR_{A}^{Y}=\prod_{q}^{M}\prod_{i}^{P}\int dr_{i}^{q}\delta ({\bar{r}}^{q})$, and the normalization factor is $N^{Y}=\prod_{q}{\left(P/2\pi \lambda_{q}^{2}\right)}^{3P/2}$. 

Although it is not feasible to obtain a converged result of the partition function (Equation (6)) for a condensed phase system, the ratio of Equation (5) can be reasonably precisely determined using Zwanzig’s free energy perturbation (FEP) theory [18].

(8) $$\begin{array}{ll} \frac{Q_{A}^{H}}{Q_{A}^{L}} & =\frac{N^{H}\int dS\int dR_{A}^{H}e^{-\beta V_{eff}(r_{A}^{H},S)}}{N^{L}\int dS\int dR_{A}^{L}e^{-\beta V_{eff}(r_{A}^{L},S)}}\\ & =\frac{\int dS\int dR_{A}^{L}e^{-\beta V_{eff}(r_{A}^{H},S)}}{\int dS\int dR_{A}^{L}e^{-\beta V_{eff}(r_{A}^{L},S)}} \end{array}$$

Here, we have switched the integration variable by $R_{A}^{H}=(N^{H}/N^{L})R_{A}^{L}$ [22]. In fact, the scaling factor of the normalization constants is equal to the square root of the ratio of heavy and light isotopic masses, which is employed in the bisection sampling algorithm to obtain the ratio of the bead spread for different isotopes [22]. Multiplying and dividing the Boltzmann factor of the denominator, we obtain

(9) $$\begin{array}{ll} \frac{Q_{A}^{H}}{Q_{A}^{L}} & =\frac{\int dS\int dR_{A}^{L}e^{-\beta [V_{eff}(r_{A}^{H},S)-V_{eff}(r_{A}^{L},S)]}e^{-\beta V_{eff}(r_{A}^{L},S)}}{\int dS\int dR_{A}^{L}e^{-\beta V_{eff}(r_{A}^{L},S)}}\\ & =<e^{-\beta \Delta V_{eff}^{L\to H}(A)}{>}_{L} \end{array}$$

which is the FEP expression in Equation (2).

Note that the development and application of the FEP theory [60] are among the most significant advances in the past 30 years in molecular simulations (for a special issue on this subject, see a past paper [61]). Accurate free energy difference in solvation between two molecules can be obtained using FEP, and this is particularly advantageous in the present context since the change in discretized path due to isotope exchange is relatively small in FEP standard [61], giving rise to nearly complete overlap of solvent configurational distributions. Thus, a single simulation is sufficient in PI-FEP [62]. 

### 2.3. Double Averaging

Although Equation (9) can be directly used in path integral molecular dynamics or Monte Carlo simulations, in practice, the increased computational costs severely limit the capability of sufficient sampling of phase space. This becomes a major problem for biological systems to compute kinetic and equilibrium isotope effects of enzymatic processes [21,28]. There are two main reasons for this challenge if Equation (9) was directly used. (1) For each closed path, one must perform *P* times more energy and gradient calculations than a classical trajectory, and this can be difficult when a combined QM/MM potential is used. (2) At a given set of (centroid) coordinates, one has to perform an integration over all, i.e., converged, paths to obtain a mean force for the quantized particles embedded in a bath configuration. The combination of (1) and (2) only represents effectively a single integration step (cf. 1 fs) in molecular dynamics simulation. 

To circumvent these difficulties, we first rewrite the quantum mechanical partition function by multiplying and dividing a common factor as follows

(10) $$Q_{A}^{Y}=Q_{A}^{cmfp}\frac{\int dS\int dR(\bar{r})e^{-\beta V_{eff}(r_{A}^{Y},S)}}{\int dS\int ds_{A}e^{-\beta U(s_{A},S)}\int dR(s_{A})\exp[-\frac{P}{2\lambda_{q}^{2}}\sum_{i}^{P}{\left(\Delta r_{i}^{q}\right)}^{2}]}\;$$

where $\Delta r_{i}=r_{i}-r_{i+1}$. The classical mechanics-free particle partition function $Q_{A}^{cmfp}$ is a product of a purely classical system and free particles in terms of Feynman path integral with the position expectation values equal to the classical coordinates, $s_{A}$ [19,63]:

(11) $$Q_{A}^{cmfp}=Q_{A}^{cm}Q_{FP}^{Y}=\{\int dS\int ds_{A}e^{-\beta U(s_{A},S)}\}\{N_{A}^{Y}\int dR(s_{A})e^{-\frac{P}{2\lambda_{q}^{2}}\sum_{i}^{P}{\left(\Delta r_{i}^{q}\right)}^{2}}\}\;$$

where $s_{A}$ denotes the classical position vectors of quantized particles, and $U(s_{A},S)$ is the potential energy of the system treated entirely classically. Applying the same trick of multiplying and dividing the Boltzmann factor of the classical system in the numerator, we obtain

(12) $$Q_{A}^{Y}=Q_{A}^{cmfp}\frac{\int dS\int d{\bar{r}}_{A}^{Y}e^{-\beta U({\bar{r}}_{A}^{Y},S)}\frac{\int dR({\bar{r}}_{A}^{Y})e^{-\beta \Delta \bar{U}({\bar{r}}_{A}^{Y},S)}\exp[-\frac{P}{2\lambda_{q}^{2}}\sum_{i}^{P}{\left(\Delta r_{i}^{q}\right)}^{2}]}{\int dR(s_{A})\exp[-\frac{P}{2\lambda_{q}^{2}}\sum_{i}^{P}{\left(\Delta r_{i}^{q}\right)}^{2}]}}{\int dS\int ds_{A}e^{-\beta U(s_{A},S)}}\;$$

In Equation (12), the difference potential $\Delta \bar{U}({\bar{r}}_{A}^{Y},S)$ is given below

(13) $$\Delta \bar{U}({\bar{r}}_{A}^{Y},S)=\frac{1}{P}\sum_{q}^{M}\sum_{i}^{P}\left\{U(r_{i}^{q},S)-U(s_{A}^{q},S)\right\}\;$$

Making use of the constraint that the average positions of the discretized path for each particle, i.e., the centroid positions ${\bar{r}}_{A}^{q}=1/P\sum_{i=1}^{P}r_{i}^{q}$, correspond to the coordinates of the classical system, $\left\{{\bar{r}}_{A}^{q}\equiv s_{A}^{q}\right\}$ [64,65,66,67], we find that Equation (12) is reduced exactly to the simple form as a double average [17,22,26,27,68,69]:

(14) $$Q_{A}^{Y}=Q_{A}^{cmfp}<<e^{-\beta \Delta \bar{U}({\bar{r}}_{A}^{Y},S)}{>}_{FP,\bar{r}}{>}_{U}\;$$

In Equation (14), the outer average $<\cdots {>}_{U}$ is obtained according to the potential $U(r_{A},S)$, which is of QM/MM type, and the simulation is carried out purely by classical mechanics in nuclear degrees of freedom. The inner average $<\cdots {>}_{{}_{FP,\bar{r}}}$ represents a free-particle sampling,

(15) $$<\cdots {>}_{{}_{FP,\bar{r}}}=\frac{\int dr_{P}\;\{\cdots \}\;\delta (\bar{r})e^{-(P/2\lambda^{2})\sum_{i}^{P}{\left(\Delta r_{i}\right)}^{2}}}{\int dr_{P}\;\delta (\bar{r})e^{-(P/2\lambda_{m}^{2})\sum_{i}^{P}{\left(\Delta r_{i}\right)}^{2}}}\;$$

but it is constrained to match the path integral centroid coordinates with the classical atomic positions. The physical interpretation of Equation (15) is the free energy difference (after taking natural logarithm and multiplying by −*RT*) between the real (quantum) system and a “free-particle” reference state. 

Given the expression for the quantum mechanical partition function in Equation (14), the ensemble average of the operator $\hat{O}$ can be evaluated by reweighting the classical mechanical average, a.k.a., umbrella sampling [70]:

(16) $$<\hat{O}>=\frac{<<O(s_{A},S)e^{-\beta \Delta \bar{U}({\bar{r}}_{A}^{Y},S)}{>}_{FP,\bar{r}}{>}_{U}}{<<e^{-\beta \Delta \bar{U}({\bar{r}}_{A}^{Y},S)}{>}_{FP,\bar{r}}{>}_{U}}\;$$

Other practical methods can be used to compute these averages such as centroid molecular dynamics [71,72,73,74] and ring-polymer molecular dynamics [75]. The double averaging approach, which is also called quantized classical path method [17,26,68,76], has been applied to model systems like single-electron-in-hard-spheres [27] as well as real systems like enzymes [77]. In PI-FEP, we separate the PI quantum average into hybrid classical molecular dynamics and free-particle path-integral simulations. The theory is exact, but the separation is especially useful because it first allows the solute–solvent-protein (system-bath) conformation to be extensively sampled (outer average), which is then followed by free energy simulation (inner average) to account for NQE. The latter free-particle sampling also results in faster convergence by avoiding the ring-polymer distribution from being trapped in local minima. This is because each new path (beads distribution) is generated independently from the previous distribution and they are 100% accepted (i.e., configurations explored) from the FP-probability density. 

### 2.4. Equilibrium Isotope Effects

The PI-FEP method presented in this article has been introduced previously for studying kinetic isotope effects of enzyme-catalyzed reactions [17], but its application to computing isotope fractionation factor is new. The equilibrium isotope fractionation factor can be expressed in terms of the ratios of partition functions:

(17) $$\alpha_{A/B}=\frac{R_{A}}{R_{B}}=\left(\frac{Q_{A}^{H}}{Q_{A}^{L}}\right)\left(\frac{Q_{B}^{L}}{Q_{B}^{H}}\right)\;$$

Equation (17) is equivalent to evaluating the relative probabilities between the heavy and light isotopes of two different compounds *A* and *B*, or the same compounds in different phases. This can be conveniently accomplished using FEP by perturbing the atomic mass from one isotope into another, e.g., *L* to *H* [17]. Note that a number of recent studies have occured with similar, if not identical, approaches for computing isotope effects [78,79,80,81,82]. The key concept in our method is that only one path-integral simulation of a given isotopic substance is needed to yield the free energy difference, i.e., the ratio of the partition function, of another isotope, resulting in good precision for calculations of isotope effects [17,69]. Consequently, not only H/D primary kinetic isotope effects (which are relatively large and easily treated) can be computed, but also heavy atom and secondary isotope effects (difficult in computation because they are close to 1) can be determined accurately [62]. This method is called path-integral free energy perturbation (PI-FEP) theory. 

To compute $\alpha_{A/B}$ (Equation (17)) using Equation (16), two simulations (X=A,B) are needed to obtain the ratio of the partition functions for a system with light-to-heavy isotope substitution:

(18) $$\frac{Q_{X}^{H}}{Q_{X}^{L}}=\;\frac{<\delta ({\bar{r}}_{X}-s_{X})<e^{-\beta \Delta \Delta {\bar{U}}_{X}^{L\to H}}e^{-\beta \Delta \bar{U}({\bar{r}}_{X}^{L},S)}{>}_{FP,L}{>}_{U}}{<\delta ({\bar{r}}_{X}-s_{X})<e^{-\beta \Delta \bar{U}({\bar{r}}_{X}^{L},S)}{>}_{FP,L}{>}_{U}}\;$$

where the subscripts *FP*,*L* specify an FP-ensemble average carried out using the potential energy for the light isotope, $\Delta \bar{U}({\bar{r}}_{X}^{L},S)$ is the difference (Equation (13)) between the average potential of the *P*-discrete beads of the light particle and that of the classical (centroid) system, $\delta ({\bar{r}}_{X}-s_{X})$ requires that the centroids are constrained to classical coordinates, and $\Delta \Delta U^{L\to H}=$ $\Delta \bar{U}({\bar{r}}_{X}^{H},S)-\Delta \bar{U}({\bar{r}}_{X}^{L},S)$. 

## 3. Computational Details

To illustrate the present dual QM/MM strategy for computing equilibrium isotope fractionation factor using the PI-FEP strategy [17], we chose the small organic solutes acetone and acetaldehyde as model compounds for the ^18^O enrichment of carbohydrates in water. Thus, a total of three separate simulations are needed, the isotope exchange of acetone, acetaldehyde, and water in aqueous solution. A main goal is to test the convergence of solvent configurational sampling through double averaging. Thus, the semi-empirical parameterized model 3 (PM3) Hamiltonian [55] was used to represent the solute molecule. The accuracy of the computed absolute fractionation factor can certainly be improved if a more accurate QM model such as a DFT representation of the solute were used, but we have not further explored these choices. It would be of interest to perform a systematic investigation in future studies. The solvent molecules were described by the TIP3P three-point charge model for water [83]. 

Each solute molecule is dissolved in a cubic box of about 34 × 34 × 34 Å^3^, consisting of 1338 water molecules. Periodic boundary conditions along with particle mesh-Ewald was used to treat long-range electrostatic interactions using the isothermal-isobaric (NPT) ensemble at 25 °C and 1 atm to generate the classical trajectory for the outer averages of Equation (18). In FP path-integral sampling, a smoothing function was used to feather intermolecular interactions to zero between 13.0 and 13.5 Å since we do not expect long range effects are critical to NQE. Overall, at least 10 ns molecular dynamics simulations were discarded as equilibration for each system, followed by about 500 ps simulations for averaging. As in the past, atoms that are within two covalent bonds of the atom (oxygen) with isotope exchange are quantized and represented by 32 or 64 beads. We have previously carried out extensive tests of the convergence of FP sampling using different quasi-particles [22], and the present choice has consistently yielded converged results. In this work, we further evaluated the convergence of FPPI sampling with respect to the number of quasi-particles used to represent each quantized particle path. We have also tested the effect of different intervals for saving classical coordinates on the computed nuclear quantum effects. 

A practical issue in path integral simulations is to obtain converged results within a tolerable amount of simulation time, especially at a precision needed for computing isotope effects. To this end, we have employed a bisection sampling technique [22,69], based on the approach developed by Ceperley for free particle sampling in which the initial and final beads are not connected [59,84]. In our implementation, the bisection sampling was performed similar to the original algorithm [59,84], but we enforced the first and last beads to be identical to enclose the ring-polymer. We then impose the condition that the centroid position matches the corresponding atomic coordinates by rigid-body translocation [22,69]. Since free-particle distribution is known exactly at a given temperature, each ring-polymer distribution is generated according to this distribution and thus 100% accepted. Furthermore, in this construction, each new configuration is created independently, starting from a single initial bead position, allowing the ring-polymer configurations to move into completely different regions of configurational space. This latter point is especially important to enhance convergence to avoid being trapped in a local minimum of the classical potential in path integral sampling. Using this sampling technique, the PI-FEP method has been applied to a range of condensed-phase and enzymatic systems [17,22,50,62,69,77,85,86].

In the bisection sampling scheme, the perturbed heavy isotope positions are related to the lighter ones by

(19) $$\frac{r_{i,L}^{q}}{r_{i,H}^{q}}=\frac{\lambda_{q_{L}}^{}\theta_{i}^{q}}{\lambda_{q_{H}}^{}\theta_{i}^{q}}=\sqrt{\frac{m_{q,H}}{m_{q,L}}};i=1,\;2,\;\cdots ,\;P;q=1,\;2,\;\cdots ,\;M\;$$

where $r_{i,L}^{q}$ and $r_{i,H}^{q}$ are the coordinates for bead *i* of the corresponding light and heavy isotopes of the classical atom *q*, $m_{q,L}$ and $m_{q,H}$ are the masses for the light and heavy nuclei, and $\theta_{i}^{q}$ is a position vector in the bisection sampling scheme [22]. In our PI-FEP (or PI-FEP/UM) simulation scheme, the position vectors for the perturbed beads, e.g., the heavy isotope, are made identical to the reference (i.e., light) isotope distribution [17]. As a result, bead positions of the perturbed mass are solely determined by the square root of mass ratio (Equation (19)), reflecting the quantum mechanical spread of the particle density.

All simulations have been carried out using CHARMM (version 42b1, Harvard University, Cambridge, MA, USA, www.charmm.org) [87], in which all techniques described in this article have been implemented [17].

## 4. Results and Discussion

First we consider the convergence of path integral simulations for computing the ratio of the quantum mechanical partition function (Equation (18)) with respect to the number of beads to represent the discretized paths or ring polymers. Acetaldehyde and water in aqueous solution are used in this test. We quantized all atoms that are within two covalent-bond range, including all three atoms of water, and all non-hydrogen atoms and the hydrogen atom bonded to the carbonyl carbon (four atoms). This has been a scheme we adopted in the past for studying kinetic isotope effects of enzymatic reactions, which is kept here. Since we do not know the exact value of the quantum mechanical partition function in the present case using the combined QM/MM-PM3/TIP3P potential, we can only check the asymptotic behavior with increasing number of beads, which approaches the exact quantum result when P→∞. For each system, we used a 100 ps trajectory from a Newtonian MD simulation, during which atomic coordinates were saved in every 100 fs (1 fs integration step is used in all MD simulations). A total of 1000 configurations were saved. Then, for each classical configuration, FPPI averaging was performed, in which the first 10 ring-polymer configurations were discarded. We note that there is no particular reason for not including these configurations since we do not need to carry out an equilibration as the FP ring-polymer configurations are generated according to their quantum mechanical distribution. Then, 200 configurations were used for averaging (the inner average in Equation (18) for each classical, i.e., centroid configuration). The results are reported by averaging over all solvent configurations (the outer, CM average). Thus, a total of 200,000 path integral configurations were included in averaging. This translates to 210,000 × P QM/MM energy calculations in each case, where *P* = 2*n* and *n* = 2 to 7 with an increment of 1 (the power over 2 is required in the bisection sampling scheme). 

**Figure 1:**
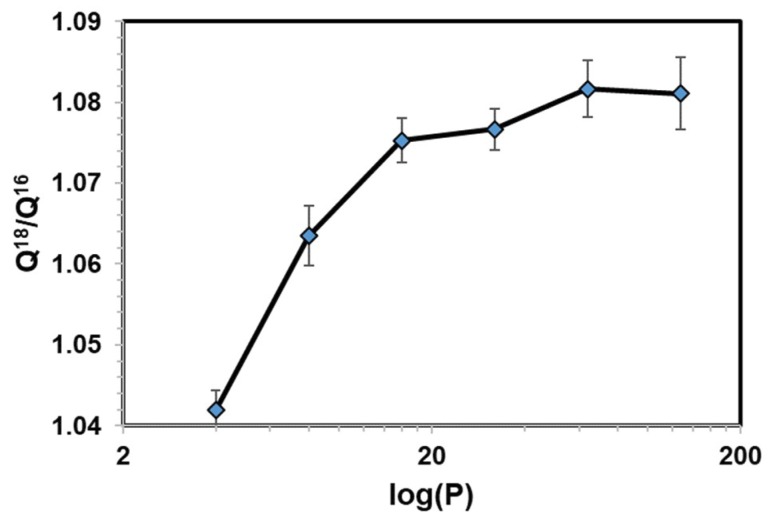
Computed ratio of ^18^O and ^16^O partition functions (Q^18^/Q^16^) versus the number of quasi-particles *P* (= 4, 8, 16, 32, 64, and 128) used in path integral simulations of water in aqueous solution. Error bars were obtained from 10-block averages over the entire simulation.

**Figure 2:**
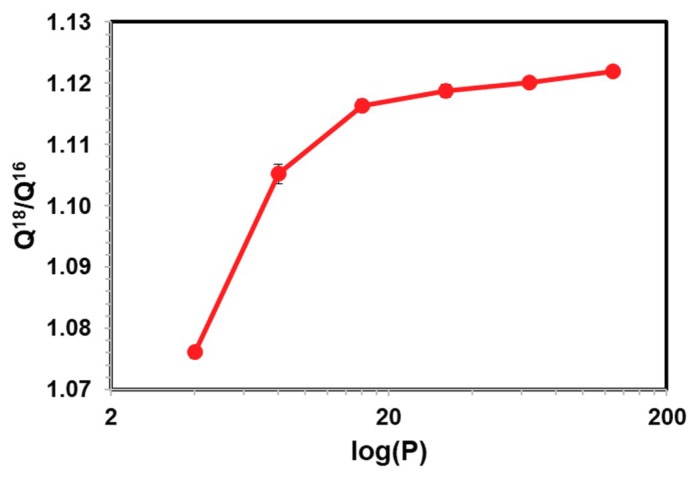
Computed ratio of ^18^O and ^16^O partition functions (Q^18^/Q^16^) versus the number of quasi-particles P used in path integral simulations of acetaldehyde in aqueous solution. For most points, the error bars are small and hidden behind the data symbols.

**Figure 3:**
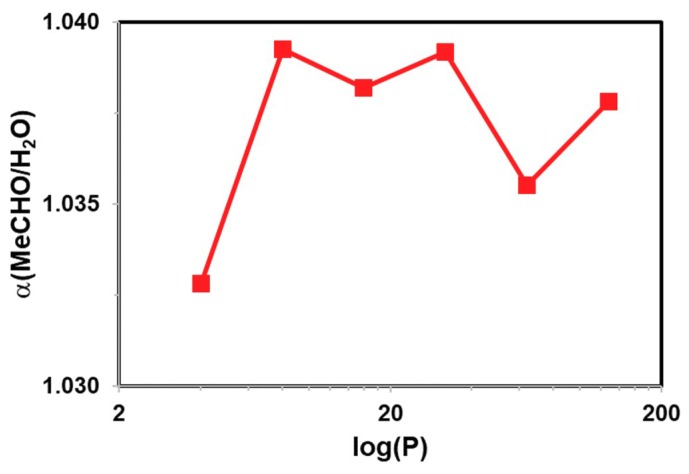
Computed ^18^O/^16^O equilibrium isotope fractionation for acetaldehyde in water versus the number of quasi-particles *P* used in path integral simulations.

Figure 1 and Figure 2 display, respectively, the ratio *R* = [^18^O]/[^16^O] for water and for aldehyde, an alchemical equilibrium constant by mutating the naturally most abundant isotope ^16^O into ^18^O through free energy perturbation. The equilibrium constants are greater than unity because the compounds containing the lighter isotope have higher vibrational frequencies and zero-point energy than the heavy isotopologues. In both cases, the computed *R* quickly and monotonically approaches its average value. In comparison to the average values obtained using 128 beads, the deviations are 0.5 (0.5)%, 0.4 (0.3)%, and 0.05 (0.2)% for water (acetaldehyde) using 16, 32, and 64 beads, respectively. Clearly, in both systems, the use of four and eight beads is not sufficient for path integral convergence (having absolute errors of about 4% and 1.5%, respectively, in the two cases). We find that the use of 32 beads offers a good balancing of the computational costs and precision, consistent with previous studies of kinetic isotope effects in enzymatic reactions [77,88]. Interestingly, the estimated isotope fractionation factors of acetaldehyde relative to water, i.e., equilibrium enrichment of the aldehyde in aqueous solution, are relatively invariant with respect to different number of beads (Figure 3), although the smallest value, *P* = 4, tested has relatively larger deviations than the rest.

**Figure 4:**
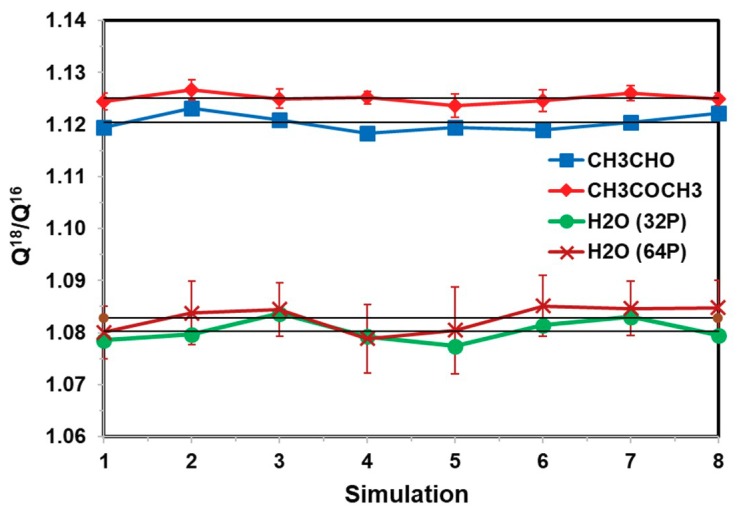
Computed ^18^O/^16^O equilibrium isotope fractionation for water, acetaldehyde, and acetone in water from eight sequential separate averages, in which the solvent configurations (outer average of Equation (18)) are saved ranging from every 1 to 100 time steps. For each configuration generated from Newtonian molecular dynamics at 25 °C and 1 atm, free-particle path-integral simulations are carried out to sample 210 random closed paths, of which the first 10 are discarded. For each block, 1000 configurations are included. Thus, each point in the figure corresponds to a FPPI averaging over 200,000 paths. 32 quasi-particles for acetaldehyde and acetone and 64 beads are used to represent each discretized path. The error bars for each point are estimated from 10 separate block-averages; those for acetaldehyde are smaller than the size of the symbols displayed, whereas those for water from 32-bead simulation are not shown for similarity to 64-bead. The fluctuations of the block averages mainly feature solvent configuration fluctuations.

We next examine the effect of solvent sampling on the computed (ratio of) NQE. Figure 4 displays the computed Q^18^/Q^16^ ratios for water, acetaldehyde, and acetone in water, obtained over 1000-configuration blocks saved at different intervals of MD steps, ranging from every 1 fs to 100 fs (1 fs integration steps). For each configuration, 200 random closed paths after discarding the first 10 configurations, represented by 32 quasi-particles for acetaldehyde and acetone and 64 for water, were used in the free-particle path-integral averaging. Thus, each point in Figure 4 represents an average over 200,000 path integral configurations (12.8 to 25.6 M QM/MM energy calculations to obtain the heavy to light isotope ratio). These configurations were divided into 10 separate blocks, from which standard deviations were determined based on these block-averages. The standard deviations for the carbonyl compounds were small in Figure 4. The variations of these eight separate averages, which were used together to determine the final isotope fractionation factor discussed next, reflect the dynamic fluctuations of the solvent configurations. For these small molecules in a rather homogeneous solvent, the fluctuations are relatively small, although they are certainly noticeable and cannot be neglected.

The computed equilibrium isotope enrichments for acetaldehyde and acetone, relative to its abundance in water are given in Table 1. In both cases, the ^18^O isotope is enriched at the carbonyl position relative to that of water. As is well-known, carbonyl compounds can rapidly undergo isotope exchange with water, especially for unhindered carbonyls, through formation of hydrates. This reaction has been attributed to be a primary biochemical factor responsible for the ^18^O enrichment found in carbohydrates such as celluloses in trees. As a result, the much higher ^18^O enrichment in CO_2_ introduced in carbon fixation by RuBisCO is quickly lost through the carbonyl-hydrate equilibrium from small sugar compounds. An experimental measurement of the ^18^O enrichment of acetone in water yielded a fractionation factor of 1.027 [6], coincidentally the same as the average enrichment in trees. We were not able to find an experimental isotope fractionation value for acetaldehyde. The computed value of *δ*^18^O about 39‰ for acetone is in reasonable agreement with experimental data, in view of the semi-empirical potential energy function used. The present semi-empirical PM3 method is perhaps among the fastest electronic structure models, allowing for adequate condensed-phase sampling. However, the harmonic vibrational frequencies for carbonyl groups are about 14% higher than experimental data (1979 cm^−1^ from PM3 vs. 1731 cm^−1^ from experiments for acetone), contributing to the absolute errors in the computed isotope effects. The computational accuracy may be improved by using a higher level of theory that can provide a good description of molecular vibrations.

We also performed calculations for the site-specific isotopic exchange of a deuterium at the C1 carbon of acetaldehyde for a proton on the methyl group. The fractionation ratio of deuterated-C2 isotopomer over that at the C1 position is related to the equilibrium constant *K* for the following isotopic exchange

$${\mathrm{CH}}_{3}\mathrm{CHO}+{\mathrm{CH}}_{3}\mathrm{CDO}⇆{\mathrm{CH}}_{2}\mathrm{DCHO}+{\mathrm{CH}}_{3}\mathrm{CHO}$$

In this case, *K* is given by the ratio of the two ratios of D/H partition functions for the H-to-D replacements in CH_2_DCHO and CH_3_CDO, and we have computed both pairs in the gas phase and in aqueous solution. The computed results are listed in Table 2. For the D/H ratio, the values are much greater than those of oxygen isotopes because of the large mass difference, leading to greater difference in vibrational frequency and zero-point energy. Both in the gas phase and aqueous solution, we found that the methyl position is enriched by more than 200‰ per hydrogen of the methyl group. If the symmetry factor of three equivalent positions is considered, the present PM3/TIP3P model yields a predicted enhancement of about 700‰ at the C2 position over that at the C1 position. Interestingly, there is little solvent effect on this isotope fractionation factor.

## 5. Concluding Remarks

We have described the application of a dual QM and MM approach for computing equilibrium isotope effects, (1) to treat the potential energy surface by a QM/MM potential, and (2) to partition the quantum mechanical statistical mechanical ensemble average into a double classical mechanical and quantum mechanical averaging. These partitions allow the potential surface to be properly represented such that the solute part is free of nuclear quantum effects for path integral simulations, whereas effects of the solvent environment is represented by an effective potential that can reproduce experimental properties of the liquid. The double-averaging simulation approach has the advantage of sampling solvent configurations, using straightforward molecular dynamics simulations for simple solutions as illustrated in this study, or applying various enhanced sampling techniques for complex systems. Importantly, the use of a free particle reference state, coupled with bisection sampling, in path integral simulations helps achieve fast convergence, (a) to avoid being trapped in local minima, and (b) to generate closed path distributions according to a known distribution. Furthermore, the precision needed for determining isotope fractionations is obtained through free energy perturbation theory to alchemically mutate the closed paths for one isotope into another, enforcing the correspondence of the centroid positions to those of classical coordinates. The PI-FEP approach is used to determine the isotope fractionation factor of oxygen in carbonyl compounds as a model for the observed isotope enrichment in tree rings. Additionally, the site-specific fractionation factor of D/H isotopomers of acetaldehyde has been estimated. The present method may be applied to systems of geochemical and ecohydrological relevance to gain an understanding of isotope fractionation.

## Figures and Tables

**Table 1:** Computed ratio of partition functions and ^18^O enrichments of CH_3_CHO and CH_3_COCH_3_ in aqueous solution at 25 °C and 1 atm using the PM3/TIP3P QM/MM potential.

	H_2_O	CH_3_CHO	CH_3_COCH_3_
Q^18^/Q^16^	1.0828 ± 0.0024	1.1204 ± 0.0014	1.1251 ± 0.0007
$\delta^{18}O$	1	0.035	0.039

**Table 2:** Computed ratio of ^2^H/^1^H partition functions and equilibrium isotope fractionation factor of CH_3_CHO in the gas phase and in aqueous solution at 25 °C and 1 atm using the PM3/TIP3P QM/MM potential.

	CH_3_C[D/H]O		[D/H]CH_2_CHO	
	Gas	Aqueous	Gas	Aqueous
Q^2^/Q^1^	19.20 ± 0.36	19.63 ± 0.98	23.72 ± 0.86	24.01 ± 1.12
$\alpha_{C2/C1}$	1	1	1.24	1.22
